# New Appliances and Things Medical

**Published:** 1902-04-26

**Authors:** 


### NEW APPLIANCES AND THINGS MEDICAL.

[We shall be glad to receive at our Office, 28 & 29 Southampton Street, Strand, London, W.C., from the manufacturers, specimens of all new preparations and appliances which may be brought out from time to time.]

### ROBB'S NURSERY BISCUITS.

(Alexander Robb and Company, 79 St. Martin's Lane, London, W.C.)

These biscuits, which were the invention of the late Dr. Golding, have been solely manufactured by the above firm for over 70 years. As an indication of the manner in which this form of infant food has been appreciated by many of our foremost physicians, it may be mentioned that it has been supplied to four generations of our Royal Family. The special process of manufacture which is employed in the preparation of these buscuits insures that they will be well tolerated by the delicate stomachs of infants and young children, and if judiciously added to the milk which constitutes the basis of their dietary, food elements of the greatest value for the nourishment and development of the infant will be provided. The feeding of infants is a matter of such supreme importance, that a food such as Robbs' biscuits, which has survived the most extended and searching trial, should be welcomed in every nursery as a reliable and valuable stand-by.

### KARNOID.

(The Karnoid Laboratory, Caterham, Surrey.)

The meat-powders which pass under the name of Karnoid are nutritive preparations of an entirely new kind. From a scientific point of view they present features of the very greatest interest, for heretofore no means have been devised by experts for preserving animal flesh in the desiccated form without so altering its physiological properties as to render it a very debatable question whether they preserve in the least degree the valuable nutritive qualities which are peculiar to fresh-meat juice. Raw-meat juice may of course be preserved by the addition of saline substances, but for meat to be kept indefinitely in the dried condition, and to be capable of assuming its original physical and chemical condition by the mere addition of water, is a very great scientific achievement. The meat-powders which are at present prepared in this interesting form by the Karnoid process are beef, chicken, and turtle. These desiccated powders are supplied in glass bottles with removable metal lid, and further protected from atmospheric influences by a hermetically sealed cork. The bottles are graduated, so that a quantity of the powder sufficient for making a teacupful of soup may be measured with accuracy. The resulting soup may be strained or unstrained according to requirement or taste. By preparing this beef, chicken, or turtle soup according to the directions a nutritive fluid is obtained which has many advantages over ordinary meat-extracts, or indeed over soups made by the ordinary processes. The flesh from which the powders are prepared have never been submitted to the destructive action of heat, and retain the important properties which belong to fresh, uncoagulated animal albumin. They should be valuable for many of the purposes for which meat is used.

### CORONATION SOAP.

(Edward Cook and Co., Limited, East London Soap Works, Bow, London, E.)

In commemoration of the Coronation year, Edward Cook and Co., the well-known soap manufacturers, are supplying the public with an entirely new variety of toilet soap, which is to be known as "the Coronation." We have examined this soap and cannot bestow higher praise upon than to express our opinion that it deserves to rank with that eminently pure and popular soap, namely, Cook's Riviera. The new soap is practically colourless, and to the mode of scenting very great attention has been paid. The scent is of a sweet and delicate blend, neither strong nor pungent, and thoroughly adapted for toilet use by the great majority of Messrs. Cook's *clientèle*, who appreciate the refinements of their exclusive manufacture.

